# The Effect of Intrahippocampal Insulin Injection on Scopolamine-induced Spatial Memory Impairment and Extracellular Signal-regulated Kinases Alteration

**DOI:** 10.32598/bcn.9.10.165

**Published:** 2019-01-01

**Authors:** Ahmad Jahanmahin, Zahra Abbasnejad, Abbas Haghparast, Abolhassan Ahmadiani, Rasoul Ghasemi

**Affiliations:** 1. Neuroscience Research Center, Shahid Beheshti University of Medical Sciences, Tehran, Iran.; 2. Neurophysiology Research Center, Shahid Beheshti University of Medical Sciences, Tehran, Iran.; 3. Department of Physiology, School of Medicine, Shahid Beheshti University of Medical Sciences, Tehran, Iran.

**Keywords:** Alzheimer Disease, Cholinergic neurons, Scopolamine, Mitogen-Activated Protein Kinases, Caspase-3, Apoptosis

## Abstract

**Introduction:**

It is well documented that insulin has neuroprotective and neuromodulator effects and can protect against different models of memory loss. Furthermore, cholinergic activity plays a significant role in memory, and scopolamine-induced memory loss is widely used as an experimental model of dementia. The current study aimed at investigating the possible effects of insulin against scopolamine-induced memory impairment in Wistar rat and its underlying molecular mechanisms.

**Methods:**

Accordingly, animals were bilaterally cannulated in CA1, hippocampus. Intrahippocampal administration of insulin 6 MU and 12 MU in CA1 per day was performed during first 6 days after surgery. During next four days, the animal’s spatial learning and memory were assessed in Morris water maze test (three days of learning and one day of retention test). The animals received scopolamine (1 mg/kg) Intraperitoneally (IP) 30 minutes before the onset of behavioral tests in each day. In the last day, the hippocampi were dissected and the levels of MAPK (mitogen-activated protein kinases) and caspase-3 activation were analyzed by Western blot technique.

**Results:**

The behavioral results showed that scopolamine impaired spatial learning and memory without activating casapase-3, P38, and JNK, but chronic pretreatment by both doses of insulin was unable to restore this spatial memory impairment. In addition, scopolamine significantly reduced Extracellular signal-Regulated Kinases (ERKs) activity and insulin was unable to restore this reduction. Results revealed that scopolamine-mediated memory loss was not associated with hippocampal damage.

**Conclusion:**

Insulin as a neuroprotective agent cannot restore memory when there is no hippocampal damage. In addition, the neuromodulator effect of insulin is not potent enough to overwhelm scopolamine-mediated disruptions of synaptic neurotransmission.

## Highlights

- Scopolamine treatment not only deteriorates spatial learning and memory but also elevates animals swimming speed and suppresses hippocampal extracellular signal-regulated kinases (ERK) activity.
- In contrast to most models of memory loss, scopolamine-induced memory impairment is not associated with hippocampal damage.
- Scopolamine treatment disrupts spatial learning and memory without activating caspase-3, c-Jun N-terminal kinase, and p38 mitogen-activated protein kinases.
- Insulin is not always protective against memory loss; intrahippocampal insulin could not be potent enough to prevent scopolamine-induced spatial memory impairment.
- Intrahippocampal insulin could not prevent scopolamine-mediated hippocampal ERK suppression.

## Plain Language Summary

This study was all about to investigate whether insulin therapy is useful in all brain disturbances. According to the results, if the brain function, including learning and memory is disturbed transiently by scopolamine administration, insulin treatment will not protect it. These results imply that although insulin is a very useful treatment, in some situations such as transient blockade of cholinergic neurons it is not protective.

## 1. Introduction

Although brain was initially considered as an insulin-independent tissue, discovery of insulin and Insulin Receptors (IRs) within various brain structures changed scientists’ view in this regard (Havrankova, Roth, & Brownstein, 1978; Havrankova, Schmechel, Roth, & Brownstein, 1978). The distribution of IRs in brain structures is not omnipresent and some regions like the hippocampus have higher IR density (Hill, Lesniak, Pert, & Roth, 1986).

Because of high expression of IRs in hippocampus, insulin could have neuromodulatory and neurotrophic effects on hippocampus and its normal signaling is required for physiological functions of hippocampus such as spatial learning and memory (Ghasemi, Haeri, Dargahi, Mohamed, & Ahmadiani, 2013). Furthermore, insulin protects against different forms of spatial memory loss mediated by various insults (Ghasemi, Zarifkar, Rastegar, maghsoudi, & Moosavi, 2014a; Jiang, Zhang, Wu, Song, & Guo, 2008; Moosavi, Naghdi, Maghsoudi, & Zahedi Asl, 2007). On the other hand, brain insulin dysregulation plays an important role in the pathogenesis of neurodegenerative diseases such as Alzheimer Disease (AD) to the extent that AD is sometimes called type 3 diabetes (Ghasemi et al., 2013).

One of the characteristics of AD is the neurodegeneration of basal forebrain cholinergic neurons (Contestabile, 2011). New evidence is consistently provided indicating pharmacologic blockade of cholinergic function (Bartus, Dean, Beer, & Lippa, 1982) and pathological reduction of Acetylcholine (Ach) concentration in Cerebrospinal Fluid (CSF) of patients with dementia (Tohgi, Abe, Kimura, Saheki, & Takahashi, 1996) that clearly show a strong relationship between Ach-mediated neurotransmission and cognitive functions. Therefore, pharmacological blockade of cholinergic transmission by receptor antagonists, like scopolamine, is known as a pharmacological model of memory loss (Sarter & Bruno, 1997; Sunderland et al., 1986).

Some of the members of MAPK (mitogen-activated protein kinases) signaling pathway are signaling molecules, which are somehow embroiled in the pathogenesis of neurodegenerative diseases such as AD as well as most forms of experimental models of memory impairment (Kim & Choi, 2010). MAPKs are a group of serine threonine kinases effective in a variety of cellular activities and are divided into three main subgroups; Extracellular signal-Regulated Kinases (ERKs), Jun N-terminal Kinases (JNKs), and p38 MAPK (Jin et al., 2006). These kinases are mainly get activated in response to extra-cellular stimuli, including cellular stresses and play an important role in mediating cellular responses to those stimuli (Moosavi, Ghasemi, Maghsoudi, Rastegar, & Zarifkar, 2011). In this regard, some of these kinases are involved both in Long-Term Potentiation (LTP) induction as well as apoptosis and LTP disruption (Ramin, Azizi, Motamedi, Haghparast, & Khodagholi, 2011; Wang, Walsh, Rowan, Selkoe, & Anwyl, 2004).

Considering the protective and neuromodulator effects of insulin in the brain, and intricate roles that members of MAPK signaling pathway play in the neuronal damages and memory loss as well as memory formation, the present study aimed at identifying the molecular basis of scopolamine-induced memory loss and evaluating if intrahippocampal insulin administration can protect against this spatial memory decline and possible accompanied alterations in MAPKs activity as an neuronal damage in the hippocampus.

## 2. Methods

### 2.1. Drugs, antibodies, and reagents

Scopolamine hydrobromide (S0929) was purchased from Sigma, Western blot antibodies, including caspase-3 (9665), beta-actin (4970), phospho-P38 (9211), phospho-JNK (4671), phospho-ERK (4377), total-p38 (8690), total-JNK (9252), total-ERK (4695), and secondary HRP-conjugated (7074) were purchased from Cell Signaling Technology Company. Amersham ECL select (RPN2235) reagent kit was purchased from GE healthcare and Polyvinylidene Fluoride (PVDF) membrane was purchased from Millipore. Other reagents were obtained from ordinary commercial sources.

### 2.2. Animals

Adult male Wistar rats, weighing 250–300 g, were obtained from the animal house of the Neuroscience Research Center, Shahid Beheshti University of Medical Science. The animals were housed in Plexiglas cages with woodchip bedding in groups of 2–3 per cage at room temperature (25±2°C) under standard 12:12 hour light-dark cycle (light from 7:00 AM to 7:00 PM). Food and water were available ad libitum. The animal care was according to the National Institutes of Health (NIH) guide for the care and use of laboratory animals and all protocols and efforts were made to minimize the number of animals used and their suffering.

### 2.3. Surgery

The rats were randomly assigned in each group (n=7–10 per each group). For central microinjection of insulin, animals were anesthetized with Intraperitoneal (IP) administration of mixed ketamine (100 mg/kg) and xylazine (10 mg/kg) and were mounted into a stereotaxic frame. According to Paxinos brain atlas, stainless steel guide cannulas (22-gauge) were implanted bilaterally into dorsal hippocampi (AP-3.8, ML±2.2, and DV-2.7) (Paxinos & Watson, 2007). The cannulas were anchored to the skull using stainless screws and acrylic cement (Ghasemi, Zarifkar, Rastegar, Maghsoudi, & Moosavi, 2014b).

### 2.4. Drug administration

For intrahippocampal microinjections, a 5-μL Hamilton syringe was connected to the injection cannula through a short piece of polyethylene tube, the injection cannula was inserted 0.5 mm beyond the tip of guide cannulas. Bilateral intrahippocampal administration of insulin 6 MU (20.4 ng) and 12 MU (40.8 ng) was performed during six days starting at the day of surgery. All microinjections were carried out 0.5 μL/minute and the needle was left in the place for additional 5 minutes to minimize the flow back of the solution. From the day after, scopolamine (1 mg/kg) (for model group and model+insulin 6/12 group) or its vehicle (saline) (for control groups) was administered IP 30 minutes before each block of training.

### 2.5. Behavioral test

Spatial memory was assessed using Morris Water Maze (MWM) apparatus. This apparatus was consisted of a black circular tank (150 cm diameter, 50 cm height) filled with water (20±1°C) at a depth of 25 cm and a platform (10 cm diameter, 23 cm height) placed 2 m under beneath of water surface. In order to be invisible in black swimming pool, the platform was made with transparent fiberglass. The maze was divided into four equal quadrants (geographically Northwest (NW), Southwest (SW), Northeast (NE), and Southeast (SE) and start locations were set in each quadrant. Also, fixed immovable visual cues were provided in room (i.e. a camera on ceiling, a door, a column, bookshelves, and paper shapes stuck to walls). A Charge-Coupled Device (CCD) camera was located above the center of the maze to record rats’ motions and send data to an automated tracking system (Noldus, EthoVision-XT 11). Then, the intended parameters, i.e. latency to reach the platform, time spent in target quadrant, and the swimming speed, were obtained and analyzed by the software.

The whole protocol in MWM in the current study was performed in four days. In the first 3 days, the invisible platform was fixed in the center of SW quadrant and in each day, rats underwent four trials of learning starting in different release points. Before starting the first trial on the first day, rats were placed on submerged platform for 20 seconds. Then during each trial, the rat had 90 seconds to found the hidden platform; if rats reached the platform in given time, it had 20 seconds to rest there until the onset of next trial. Rats that could not find the platform within 90 seconds were guided to it by the experimenter. The latency to find the platform was extracted by the software. In the fourth day, retrieval test (probe trial) was performed. In this test, the platform was removed and rats were given 60 seconds time to swim and search for platform and parameters such as cumulative time spent in target quadrant and frequency of crossing over platform site and swimming speed were extracted by EthoVision XT 11 software.

### 2.6. Tissue preparation

At the last day of behavioral test and immediately after completion of probe trial tests, animals were anesthetized by CO2 inhalation and then decapitated and their hippocampi were quickly isolated on ice and transferred to liquid nitrogen and then stored at −80°C until biochemical analysis.

### 2.7. Western blot analysis

Western immunoblotting was used to determine expression and phosphorylation of targeted proteins. Accordingly, hippocampi of rats (n=3 assigned randomly to each group) were homogenized in cold Radioimmunoprecipitation Assay (RIPA) lysis buffer, including protease and phosphatase inhibitor. Then, samples were centrifuged (14000 g for 30 minutes at 4°C) and protein containing supernatants were collected. The protein concentration of samples was assessed by the Lowry method and according to the results; samples with equal amounts from each sample were boiled in 2x sample buffer for 5 minutes and then were loaded and electrophoresed in SDS-PAGE (sodium dodecyl sulfate polyacrylamide gel electrophoresis) 12% polyacrylamide gel.

Separated proteins were then transferred to PVDF membranes and after blocking in 5% Bovine Serum Albumin (BSA), then the membranes were incubated with primary antibodies (caspase-3, MAPKs, and β-actin) overnight at 4°C. After washing, the membranes were incubated with Horseradish Peroxidase (HRP)-conjugated anti-rabbit antibody for one hour at room temperature. Immunoreactive bands were visualized using chemiluminescent detection (ECL select) kit. Finally, the radiographic films were scanned and densitometric analysis of bands was implemented by ImageJ software.

### 2.8. Data analysis

The data obtained from three days of learning experiments were analyzed by repeated measure. The probe test data as well as molecular data were analyzed by 1-way Analysis of Variance (ANOVA). Tukey post-hoc test was used for multiple comparisons. All results were expressed as Mean±SEM. In all statistical comparisons, P<0.05 was considered as the level of significance.

## 3. Results

### 3.1. Spatial learning and memory test

**Figure 1.**
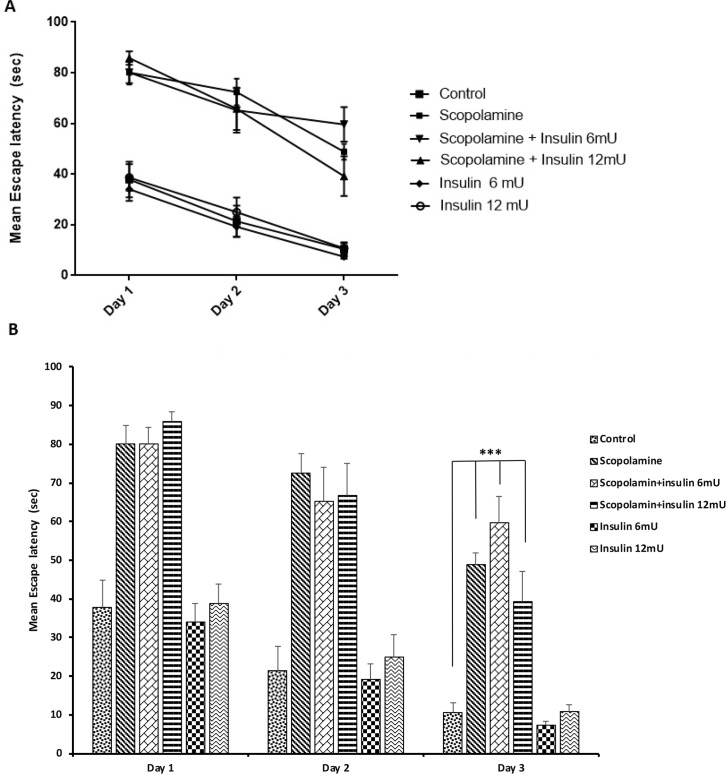
The effect of vehicle, scopolamine and or insulin treatment on water maze spatial learning and memory A) The learning patterns of the animals treated by vehicle, scopolamine, and or insulin during training sessions; and B) The escape latency to the hidden platform during days 1–3 of training. Data are expressed as Mean±SEM. *** P<0.001 represents the significant difference between control and other groups in each day.

Figure 1 shows the effect of scopolamine and or insulin (6 or 12 mU) administration on escape latency to reach the hidden platform. It is evident that a negative linear correlation exists between escape latency and training days in all groups (the relative changes of escape latencies on the day 2/the day 1 and the day 3/the day 1), indicating that all groups have learnt the platform location. However, scopolamine administration significantly slowed down the learning capability. Figure 1A shows the learning pattern of animals in all groups. Repeated measures analysis of escape latency in three training days showed that a significant difference between the groups (F_5,41_=35.308; P<0.001).

Post hoc Tukey test following repeated measures analysis revealed that escape latency in the scopolamine receiving group was significantly greater than that of the vehicle receiving group (P<0.001). This post hoc test also showed a significant difference between the control group and the groups receiving 6 or 12 mU insulin (CA1 daily for six days) prior to the initiation of scopolamine treatment and the MWM test (P<0.001). Figure 1B shows escape latency to find hidden platform during three days of training. To compare the behavior of rats in different days of training, 1-way ANOVA was used, and the results demonstrated a significant difference between groups in all the days (Day 1: F_5,41_=23.417, P<0.001; Day 2: F_5,41_=15.830, P<0.001; Day 3: F_5,41_=26.468, P<0.001). Post hoc Tukey test following 1-way ANOVA showed that scopolamine significantly increased escape latency in all training days; while insulin treatment in doses of 6 and 12 mU could not prevent the impairing effect of scopolamine.

**Figure 2.**
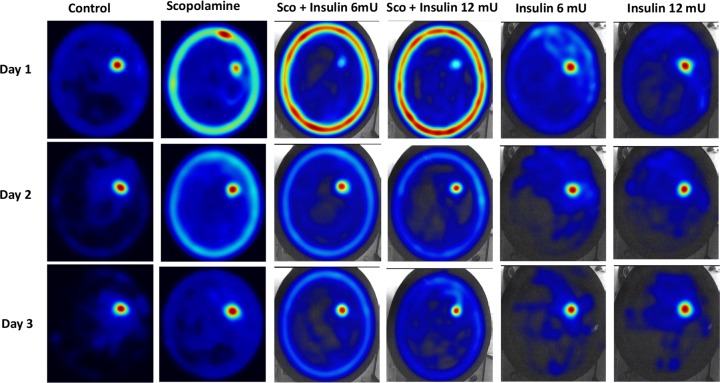
Superimposed maps of animal’s swimming paths in each group during three days of training in the MWM These heatmap figures show a comparable superimposed map of animal’s movement paths in the MWM apparatus. As it is evident, with learning trials during 3 days, it took less time to find the platform and animals swam mainly in the vicinity of the platform, but in animals treated with scopolamine, the pattern of movement does not show such improvement. The figures are obtained by merging the pattern of all trails of animals in each day for each group (Noldus, EthoVision XT 11).

Figure 2 illustrates superimposed maps of animal’s swimming paths in each group during three days of training in the MWM. The heatmap plots clearly demonstrated that although on day 1 of training animals almost explored all the quadrants of the MWM pool to find the platform, with progression of spatial learning, the animals with normal learning capability avoid entering empty quadrants and mainly swam in the vicinity of platform (target scanning pattern). On the contrary, scopolamine treated animals did not show such focused search and their swimming pattern in succeeding days did not improve like the control groups. Furthermore, the heatmaps depict that scopolamine treatment not only increased the scape latency to reach platform, but also altered the animals’ swimming pattern.

As the figures show, blockade of muscarinic receptors causes animals to show higher thigmotaxis (swimming near the wall around the outer perimeter of the tank) behavior than the control or insulin groups. This pattern of swimming is associated with a lower chance to find the platform. Finally, as it is evident in Figure 2, that insulin neither increased focused search in scopolamine treated animals, nor reduced thigmotaxis pattern in such animals.

**Figure 3.**
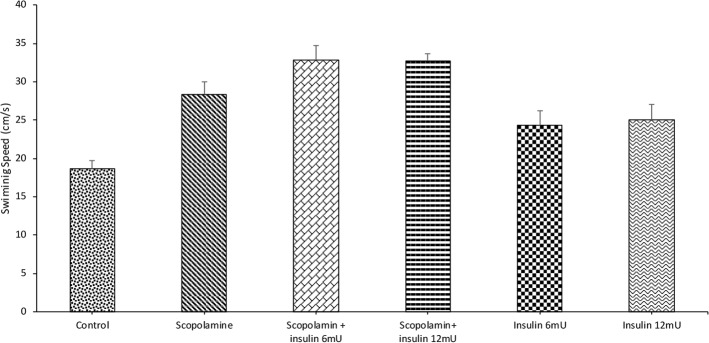
The effects of treatments on swimming speed Data are expressed as Mean±SEM. ***P<0.001 shows significant difference between the control and other groups.

Figure 3 depicts the mean swimming speed of the animals during probe test. One-way ANOVA analysis showed significant differences between the groups (F_5,41_=10.649; P<0.001). Post hoc analysis by Tukey test showed that scopolamine treatment significantly increased the swimming speed. Post hoc analysis also revealed that pre-treatment with insulin was unable to revert scopolamine-mediated increase in the swimming speed. Insulin administration by itself did not change swimming speed in the experimental group in comparison with that of the control group.

**Figure 4.**
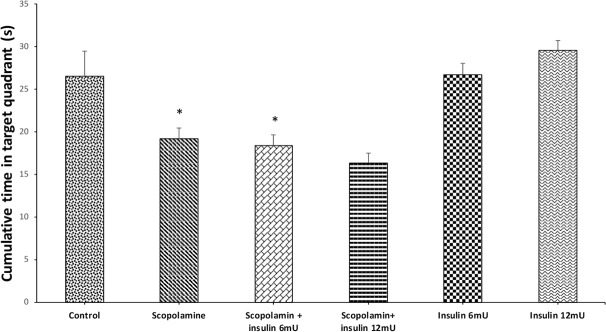
The effects of treatments on probe (retention) test Data are expressed as Mean±SEM. *P<0.05 and **P<0.01 represents significant difference between the control and other groups.

The effect of pre-probe saline, scopolamine or and insulin administration on the cumulative time percentage spent in the target zone during probe trial is depicted in Figure 4. One-way ANOVA revealed significant difference between the groups (F_5,41_=9.854; P<0.001). Results of the Tukey post hoc test showed that scopolamine treatment decreased the time percentage, which the animal swam in the target zone, and insulin could not nullify the adverse effects of scopolamine on memorizing the location of the platform.

### 3.2. Western blot results

**Figure 5.**
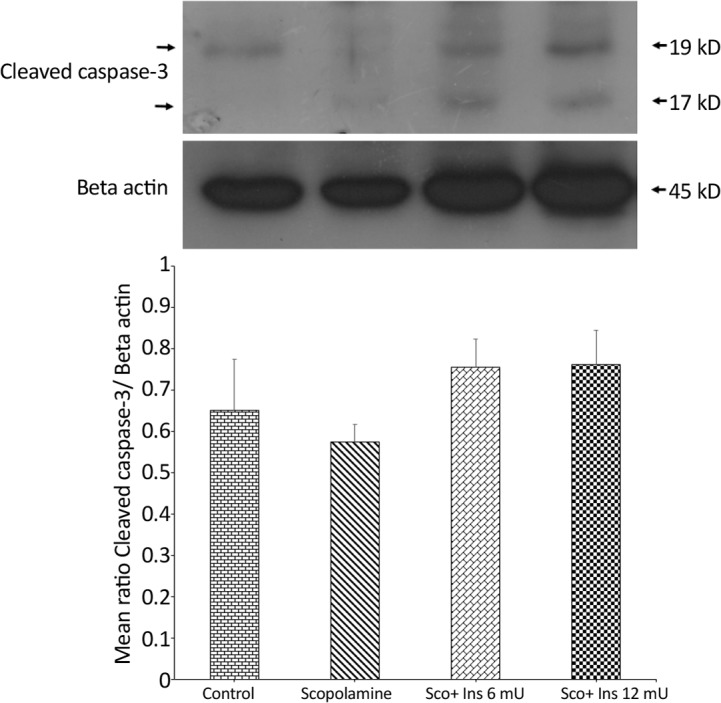
Comparative amount of caspase-3 cleavage in different groups Western blot analysis showing the effects of IP administration of saline or scopolamine on animals receiving or not receiving intrahippocampal insulin for 6 days, on hippocampal caspase-3 cleavage; immunoblots were probed with caspase-3 and β-actin antibodies. Caspase-3 activity did not show significant differences between the groups. Representative immunoblots are shown in the upper panel.

**Figure 6.**
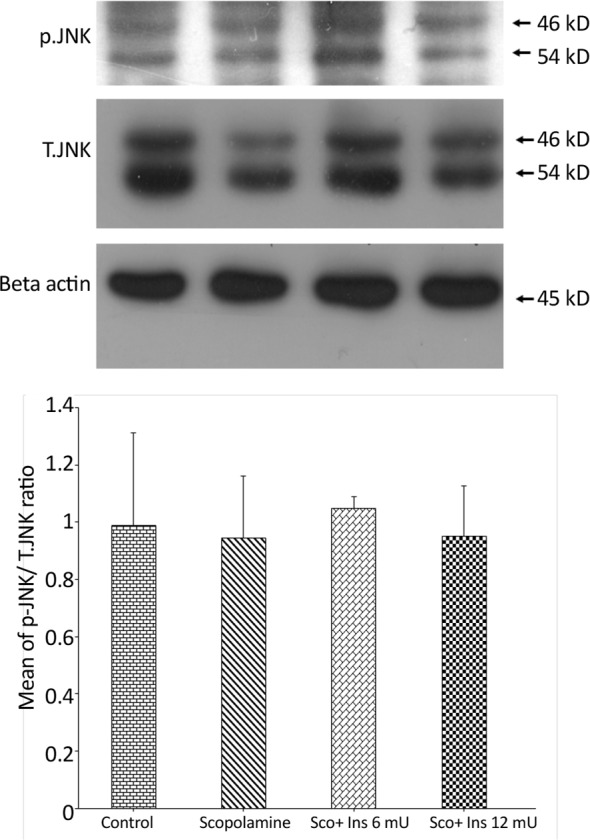
The ratio of phosphorylated to total form of JNK in the hippocampus Western blot analysis showing the effects of IP administration of saline or scopolamine on animals receiving or not receiving intrahippocampal insulin for 6 days, on phosphorylated JNK protein in the hippocampi of rats. Immunoblots were probed with antibodies against phosphorylated JNK, total JNK, and β-actin. JNK activity did not show significant differences between the groups. Representative immunoblots are shown in the upper panel.

**Figure 7.**
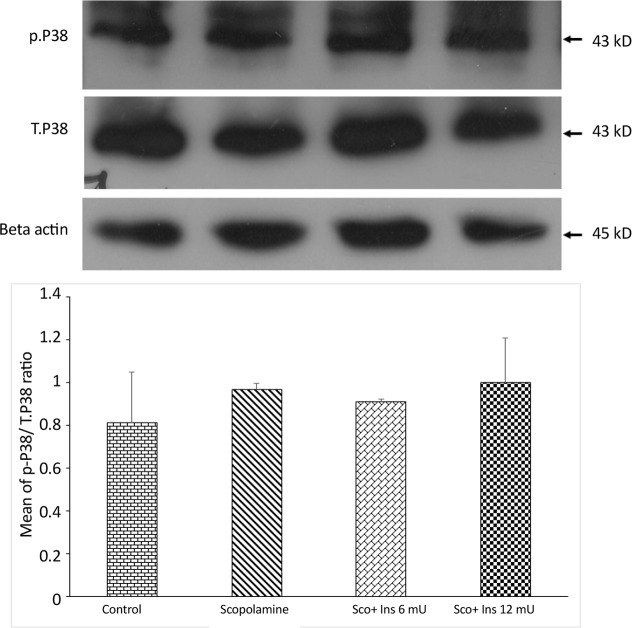
Western blot analysis on the activity of p38 as the ratio of phosphorylated to total form of p38 Western blot analysis showing the effects of IP administration of saline or scopolamine on animals receiving or not receiving intrahippocampal insulin for 6 days, on phosphorylated P38 protein in the hippocampi of rats. Immunoblots were probed with antibodies against phosphorylated p38, total p38, and β-actin. p38 activity did not show significant differences between the groups. Representative immunoblots are shown in the upper panel.

**Figure 8.**
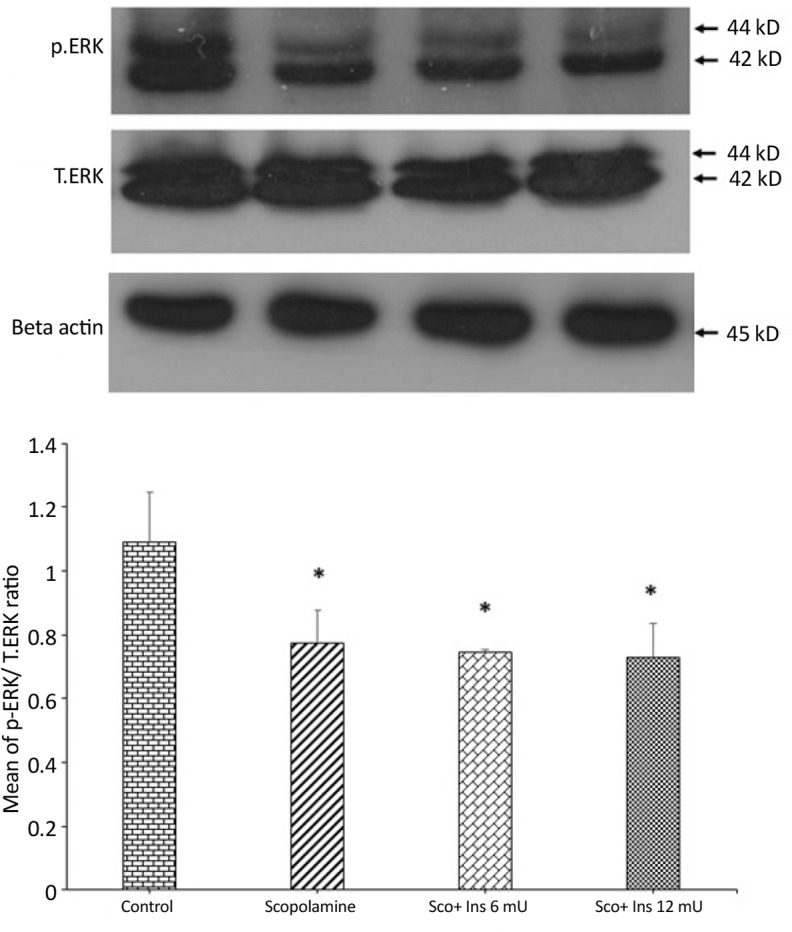
Western blot analysis on phosphorylated (activated) ERK Western blot analysis showing the effects of IP administration of saline or scopolamine on animals receiving or not receiving intrahippocampal insulin for 6 days, on phosphorylated ERK protein in the hippocampi of rats. Immunoblots were probed with antibodies against phosphorylated ERK, total ERK, and β-actin. *P<0.05 represents significant difference between the control and other groups. Representative immunoblots are shown in the upper panel.

Western blot experiments on the hippocampi of animals was performed to assess the effect of scopolamine and intrahippocampal insulin plus IP scopolamine on caspase-3 cleavage and MAPKs activity and the results are depicted in Figures 5 to 8. In order to examine if behavioral alterations were associated with hippocampal apoptosis, hippocampal lysates were probed with antibody against activated (cleaved) caspase-3 (as an indicator of apoptosis). This antibody detected two bands at 19 and 17 kDa and the comparative amount of caspase-3 cleavage (normalized to beta actin) in different groups are illustrated in Figure 5. One-way ANOVA analysis showed no significant difference between groups (F_3,8_=1.124; P=0.3953).

To explore any correlations between behavioral alterations and the activity MAPK members in the current study, the amounts of p38, JNK, and ERK phosphorylation were assessed by Western blot analyses. Figure 6 depicts the ratio of phosphorylated to total form of JNK in the hippocampus. The antibody against phosphorylated JNK detected two bands at 46 and 54 kDa. Oneway ANOVA analysis showed no significant difference between the groups (F_3,8_=0.05001; P=0.9841).

Figure 7 shows the results of Western blot analysis on the activity of p38 as the ratio of phosphorylated to total form of p38. The antibody against phosphorylated p38 detected a band at 43 kDa. One-way ANOVA analysis showed that like JNK and caspase-3, scopolamine did not affect hippocampal p38 activity significantly (F_3,8_=0.2716; P=0.8993). Figure 8 shows the results of Western blot analysis on phosphorylated (activated) ERK. The antibody against phosphorylated ERK detected two bands at 42 and 44 kDa. One-way ANOVA analysis showed significant difference between the groups (F_3,8_=7.631; P=0.0099). Post hoc analysis with Tukey test revealed that scopolamine treatment suppressed ERK activation, while no dose of intrahippocampal pre-treatment with insulin prevented scopolamine-induced decrement of ERK activation.

## 4. Discussion

The findings of the current study revealed that although scopolamine treatment significantly undermined the animals learning and memory abilities and suppressed hippocampal ERK activity, contrary to other forms of experimental models of memory loss, chronic intrahippocampal pretreatment with insulin could not restore these effects.

The scopolamine-mediated increment in latency to find hidden platform in the MWM test was significant even though rats swam at higher speed. This higher activity in the current study was consistent with other studies showing that central or peripheral administration of scopolamine induces hyperactivity in animals (Anagnostaras, Maren, Sage, Goodrich, & Fanselow, 1999; Moosavi, Khales, Abbasi, Zarifkar, & Rastegar, 2012).

The cholinergic signaling in different structures of brain such as hippocampus, frontal cortex, and or striatum seem to contribute to this hyperactivity induced by scopolamine (Klinkenberg & Blokland, 2010). It is believed that an imbalance between nicotinic cholinergic and muscarinic transmission is a factor that may cause scopolamine-induced hyperactivity occurring after blockade of muscarinic receptors (Day, Damsma, & Fibiger, 1991). Another factor, which may contribute to increased swimming speed and thigmotaxis in the MWM, is the elevated level of stress and anxiety in such animals (Gehring, Luksys, Sandi, & Vasilaki, 2015; Wolfer, Stagljar-Bozicevic, Errington, & Lipp, 1998).

The results of the current study were also in agreement with the idea that Ach neurotransmission is an essential element in learning and memory process, and blockade of muscarinic receptor by pretreatment administration of scopolamine impairs learning and memory (Deiana, Platt, & Riedel, 2011; Malikowska, Salat, & Podkowa, 2017; Moosavi et al., 2012). Hippocampus is one of the major components of the brain, which plays an important role to process temporal and spatial information, and it is shown that Ach release in the dorsal hippocampus increases during learning or exploration activities (Mitsushima, 2011). Several lines of evidence are also available showing that damaged cholinergic neurotransmission may contribute to the pathology of AD (Contestabile, 2011). It is believed that the first group of neurons injured in AD are cholinergic forebrain neurons (White-house et al., 1982). Furthermore, a recent study reported that gene expression and binding activity of muscarinic M1 receptors are hindered by beta-amyloid 1–42 peptide (Jin et al., 2009).

Because of hippocampus pivotal role in the process of spatial learning and memory and the protective effects of intrahippocampal insulin administration, we tried to investigate if insulin could protect against scopolamine-mediated memory loss. Although previous studies, including the authors`, showed that insulin could protect against memory loss (Ghasemi et al., 2014a; Moosavi et al., 2007), in the current study the same paradigm of insulin microinjection (six days in each hippocampus) was unable to restore behavioral deficits.

One difference between these studies with the current one was that in the mentioned studies, insulin administration was performed after Aβ treatment (Ghasemi et al., 2014a), but in the current study, insulin was given in a pretreatment manner. Therefore, this may explain why insulin was unable to restore memory. To test if scopolamine-induced spatial memory impairment was associated with hippocampal stress, we decided to evaluate the activity of members of MAPKs signaling pathway as well as cleavage of caspase-3 (as an indicator for apoptosis initiation) in the hippocampal lysate of the studied animals.

The current study findings showed that scopolamine treatment was not associated with the cleavage of caspase-3 when compared with the control group, indicating that in this model of memory loss, neuronal apoptosis was not initiated. Furthermore, when hippocampal JNK and p38 MAPKs activities were examined, no significant changes were detected in the activities of JNK and p38 MAPKs at the time point of the current evaluation. These kinases are typically activated when cells are stressed by various insults such as oxidative stress and inflammation and play a critical role to mediate neuronal apoptosis in such situations (Ki, Park, Lee, Shin, & Koh, 2013; Moosavi et al., 2011).

The current study outcomes strengthen the possibility that neuronal stress and or apoptosis may not be the main mechanism responsible to memory deficit in the present paradigm, and it could be just a consequence of blockade of cholinergic transmission. In support of this theory, some recent studies showed that agents with Anti-acetylcholinesterase (AChE) activities could reverse that scopolamine induced cognitive dysfunction (Seo, Lim, Kim, Lee, & Kim, 2017). The evaluated study further confirmed the current study conclusion that inhibition of cholinergic transmission plays a central role in the action mechanisms of scopolamine.

This is in contrast to most experimental models of memory decline induced by various insults such as immobilization stress (Moosavi et al., 2007), ischemia (Yi et al., 2016), β-amyloid (Zhang et al., 2015), and ibotenic acid (Kapgal, Prem, Hegde, Laxmi, & Kutty, 2016; Karthick, Periyasamy, Jayachandran, & Anusuyadevi, 2016) that all were associated with hippocampal damages.

The current study was also incompatible with some limited studies reporting that IP scopolamine treatment was coupled with hippocampal apoptosis (Balaban, Naziroglu, Demirci, & Ovey, 2016; Jahanshahi, Nickmahzar, & Babakordi, 2013). It seems that higher doses of drug (1 vs. 3 mg/kg) (Jahanshahi et al., 2013) or the higher number of injections (four days vs. three week) (Balaban et al., 2016) might be responsible for these differences. In addition, the current study results had some differences with the findings of a recent study by Moosavi, SoukhakLari, Moezi and Pirsalami, (2018) indicating that a single dose of scopolamine before retrieval session of passive avoidance test increased the activity of JNK and p38. It seems that time of drug administration (24 hours after training) and or the type of test (passive avoidance vs. the MWM) may be responsible for these differences. Furthermore, MAPK activities are time dependent and they show fluctuations in their activity. This raises another possibility to explain the discrepancy between the current study results and those of the mentioned study.

The next question is how blockade of cholinergic transmission produces such effects. It is shown that Ach receptors induce glutamate release from glutamatergic pyramidal neurons and muscarinic receptors can assist NMDA responses in the hippocampus (Khakpai, Nasehi, Haeri-Rohani, Eidi, & Zarrindast, 2012). Accordingly, one mechanism that might be involved in scopolamine adverse effects is the interruption of muscarinic receptors mediating NMDA facilitation (Markram & Segal, 1990; Ohno & Watanabe, 1998). On the other hand, several lines of evidence indicate that besides being a neuroprotective agent, insulin also plays neuromodulatory roles in the hippocampus (Ghasemi et al., 2013). Therefore, insulin potentiates the response of NMDA receptors (Liu, Brown, Webster, Morrisett, & Monaghan, 1995); this potentiation is thought to be conducted through rapid phosphorylation of NMDA subunits (NR2A, NR2B) (Christie, Wenthold, & Monaghan, 1999) as well as increment in NMDA exocytosis (Skeberdis, Lan, Zheng, Zukin, & Bennett, 2001). Therefore, although insulin could assist NMDA function and memory formation, it was unable to restore the behavioral defects observed in the current study. This can be explained by two theories; first, these results may imply that cholinergic modulation of NMDA function and memory process is stronger and more significant than it could be overwhelmed by insulin neuromodulator effects.

Second, and the more likely possibility is that unlike the neuroprotective effects of insulin, which could preserve neurons against different insults and its long-term effects, neuromodulatory effects of insulin might be transient and the effects of 6 days of insulin treatment are not lost until the time of scopolamine administration. In support of this latter explanation, in the study mentioned before about the effects of insulin on NMDA subunit phosphorylation, Christie et al. (1999) reported that 20 minutes exposure of rat hippocampal slices to 1 μM insulin increased NR2A and NR2B tyrosine phosphorylation, while as this time was extended to 60 minutes, the subunit phosphorylation was not observed.

In a series of pilot studies conducted by the authors, even when intrahippocampal insulin was administered 30 minutes before each scopolamine IP injection (60 minutes before the MWM test), it was unable to protect (data not shown). Furthermore, the current study results also showed that intrahippocampal insulin microinjection for 6 days did not enhance the learning and memory capabilities when compared with those of the control group. These results also supported the view that neuromodulatory effects of insulin could not persist long enough to counteract against scopolamine effects or potentiate memory in insulin receiving animals.

Unlike JNK and p38, which did not change by scopolamine treatment, the Western blot analysis showed that the activity of ERK declined in animals receiving scopolamine, and insulin was unable to restore this reduced ERK activity. ERK is an intricate signaling molecule that its effects range broadly from cell survival, proliferation, and differentiation, as well as synaptic plasticity and memory formation to apoptosis (Aredia, Malatesta, Veneroni, & Bottone, 2015; Roskoski, 2012). It is believed that the transient activation of ERK contributes to cell survival and memory formation, but when its activity persists, it leads to neuronal apoptosis (Ghasemi, Moosavi, Zarifkar, Rastegar, & Maghsoudi, 2015; Subramaniam & Unsicker, 2010). Since in the current study, drug treatment was performed a short time before each session of the MWM, the persistent activity of ERK was not the option of consideration.

Authors believe that reduction in ERK activity observed in the current study could be considered as an indicator of defected memory formation. In support of this view, it is demonstrated that the MWM training activates hippocampal ERK (Blum, Moore, Adams, & Dash, 1999), and IP injection of scopolamine reduces hippocampal ERK activity (Moosavi et al., 2012). However, studies on mice also indicate that hippocampal ERK does not change after single scopolamine administration (Valjent, Pages, Herve, Girault, & Caboche, 2004). This discrepancy may be due to the differences in the animals (mouse vs. rat), number of injections, and or the delay time after injection. In addition, in another study, it was observed that the activity of ERK elevated when scopolamine was administrated before passive avoidance retrieval tests (Moosavi et al., 2018).

It is suggested that this difference may imply that ERK participates in distinct stages of memory in a different manner, with a positive role in acquisition and consolidation stages, and a negative role during retrieval phase. Furthermore, some evidence also supports that stimulation of muscarinic receptors gives rise to ERK activation in the hippocampus (Jin et al., 2009) and in human coronary artery endothelial cells (Smedlund, Tano, Margiotta, & Vazquez, 2011). Since ERK plays an important role in hippocampal synaptic plasticity and memory processes, the current study finding implies that scopolamine-mediated muscarinic receptor blockage suppresses ERK activation and this suppression might be an underlying mechanism by which the scopolamine disrupts learning and memory. Finally, since insulin could not ameliorate muscarinic receptor function, it is expected that insulin could not restore hippocampal ERK suppression.

In conclusion, results of the current study reveals that mechanisms involved in the scopolamine-mediated learning and memory are quite different from those of other experimental models of memory loss mainly coupled with neuronal loss. In addition, insulin cannot suppress memory loss in such models. Furthermore, insulin neuromodulatory effects are also not potent and or long-lasting enough to compensate scopolamine adverse effects.
